# Commissioning experience with cone‐beam computed tomography for image‐guided radiation therapy

**DOI:** 10.1120/jacmp.v8i3.2354

**Published:** 2007-07-17

**Authors:** Joerg Lehmann, Julian Perks, Sheldon Semon, Rick Harse, James A. Purdy

**Affiliations:** ^1^ Department of Radiation Oncology University of California–Davis School of Medicine Sacramento California U.S.A.

**Keywords:** Cone‐beam CT, CBCT, image‐guided radiation therapy, commissioning

## Abstract

This paper reports on the commissioning of an Elekta cone‐beam computed tomography (CT) system at one of the first U.S. sites to install a “regular,” off‐the‐shelf Elekta Synergy (Elekta, Stockholm, Sweden) accelerator system. We present the quality assurance (QA) procedure as a guide for other users. The commissioning had six elements: (1) system safety, (2) geometric accuracy (agreement of megavoltage and kilovoltage beam isocenters), (3) image quality, (4) registration and correction accuracy, (5) dose to patient and dosimetric stability, and (6) QA procedures. The system passed the safety tests, and agreement of the isocenters was found to be within 1 mm. Using a precisely moved skull phantom, the reconstruction and alignment algorithm was found to be accurate within 1 mm and 1 degree in each dimension. Of 12 measurement points spanning a 9×9×15‐cm volume in a Rando phantom (The Phantom Laboratory, Salem, NY), the average agreement in the x, y, and z coordinates was 0.10 mm, −0.12 mm, and 0.22 mm [standard deviations (SDs): 0.21 mm, 0.55 mm, 0.21 mm; largest deviations: 0.6 mm, 1.0 mm, 0.5 mm] respectively. The larger deviation for the y component can be partly attributed to the CT slice thickness of 1 mm in that direction. Dose to the patient depends on the machine settings and patient geometry. To monitor dose consistency, air kerma (output) and half‐value layer (beam quality) are measured for a typical clinical setting. Air kerma was 6.3 cGy (120 kVp, 40 mA, 40 ms per frame, 360‐degree scan, S20 field of view); half value layer was 7.1 mm aluminum (120 kV, 40 mA).

We suggest performing items 1, 2, and 3 monthly, and 4 and 5 annually. In addition, we devised a daily QA procedure to verify agreement of the megavoltage and kilovoltage isocenters using a simple phantom containing three small steel balls. The frequency of all checks will be reevaluated based on data collected during about 1 year.

PACS number: 87.53.Xd

## I. INTRODUCTION

Cone‐beam computed tomography (CBCT) for patient positioning during radiation therapy represents a recent and significant advance in what is now being called image‐guided radiation therapy (IGRT). The CBCT convolution formula^(1)^ has been applied to single‐photon emission computed tomography for many years,^(2)^ but the use of a CBCT system utilizing a diagnostic X‐ray tube and a flat panel detector for radiation therapy positioning is more recent.^(^ ^3^ ^–^ ^6^ ^)^

With CBCT, a full CT scan of the patient on the treatment couch is obtained immediately before radiation delivery, with the CT scan taken and reconstructed in less than 2 minutes. The CT scan can then be automatically registered to the CT taken earlier for treatment planning to facilitate precise repositioning of the patient to the treatment machine isocenter. Development of CBCT for radiation therapy is a rapidly growing field, following the impetus for image‐guided radiation therapy.^(7)^ Several studies have already reported on technical and initial clinical aspects of kilovoltage (kV)–based CBCT.^(4–6,8–15)^

At the same time, other methods of verifying patient position before radiotherapy treatment are progressing as well. Other kV‐imaging‐based methods include a sliding gantry CT scanner installed in an existing treatment room^(16)^ and in‐line cone‐beam CT,^(17)^ where the imaging beam is mounted opposite to the treatment beam sharing the same isocenter. Megavoltage (MV) imaging‐based methods include MV CBCT^(^ ^18^ ^,^ ^19^ ^)^ and helical tomotherapy MV CT.^(^ ^20^ ^–^ ^22^ ^)^

Our institution recently installed an Elekta Synergy (Elekta, Stockholm, Sweden) system that uses a kV tube and flat‐panel amorphous silicon imager mounted orthogonally to the treatment head. This system of CBCT for image guidance has undergone significant research and development by four major collaborating centers^(^ ^5^ ^,^ ^6^ ^,^ ^9^ ^,^ ^11^ ^,^ ^23^ ^)^ and has recently been commercially released. At our institution, the linear accelerator upon which the CBCT system is mounted is in full clinical operation, following routine commissioning and calibration according to the reports of the American Association of Physicists in Medicine (AAPM) task groups 45 and 51.^(^ ^24^ ^,^ ^25^ ^)^ The Synergy system features MV portal imaging, which was clinically implemented after routine testing and calibration according to AAPM TG 58.^(26)^ Although the AAPM scientific committee is addressing this issue and an AAPM working group on imaging for treatment verification is active, no report has yet been released addressing CBCT commissioning and quality assurance (QA). We therefore developed a protocol to commission the CBCT system for clinical use. This protocol offers a series of checks and tests to ensure that the CBCT system is safe both mechanically and dosimetrically for patient use and that it performs as expected in terms of predicted repositioning. Additionally, we established a schedule of routine QA checks. Some of the checks were adopted, when appropriate, from the manufacturer's acceptance procedure; others were developed in‐house, including a daily isocenter agreement check with a new phantom.

The tests performed for the protocol reported here were based on the Elekta Synergy system, but the principles of safety and fitness‐for‐purpose are generic to all kV‐based CBCT systems and, in general, to MV‐based systems as well. Hence, this protocol could be readily adapted for use with any current commercially available system.

## II. METHODS

### A. CBCT with Elekta Synergy

To make the commissioning procedures understandable, a brief review of the process of CBCT with the Elekta Synergy follows.

**Figure 1:**
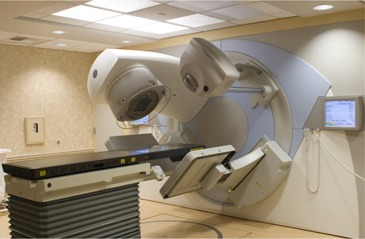
Elekta Synergy system at University of California–Davis School of Medicine.

The kV imaging system, consisting of retractable X‐ray source and amorphous silicon panel detector, is mounted orthogonally to the MV beam line of the accelerator (Fig. 1). For CBCT image acquisition, the gantry is rotated around the positioned, ready‐for‐treatment patient for a selectable angle (~200 – 360 degrees), and planar images are acquired with the kV imaging system. Volumetric image reconstruction is performed simultaneously with the acquisition to expedite the process. The reconstructed three‐dimensional geometry (localization geometry) is subsequently registered with the reference geometry (generally, the planning CT images), either manually or automatically. Automatic mode currently features two options: soft tissue and bone. For some disease sites, such as prostate cancer, the soft tissue mode is ideally suited conceptually, because the prostate often moves relative to the bones.^(^ ^27^ ^–^ ^30^ ^)^ In all other cases, the use of the bony registration was found to be the best starting point.

**Figure 2:**
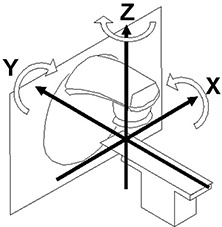
Room axes convention in the Elekta Synergy system.

Based on registration, the difference between reference and localization geometry is calculated and displayed as translation along and rotation about the three axes (Fig. 2). To bring the patient into alignment with the reference geometry, the user needs to move the patient (couch) to correct the differences. A remote auto setup tool is available.

**Figure 3:**
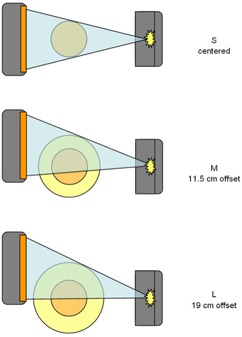
Illustration of the various settings for the width of the field of view in the kilovoltage imaging system. In the “S” setting, the kilovoltage panel is centrally aligned with the tube and the field of view of 27.67 cm (at isocenter) is centered in the middle of the patient. For the “M” and “L” settings, the panel is moved up by 11.5 cm and 19 cm respectively, resulting in partial scans and allowing larger patient diameters to be scanned.

The Elekta CBCT allows the width and the length of the kV X‐ray field to be selected. The width refers to the field of view [FOV (perpendicular to the rotational axis of the imaging system)], and the choices are small (S), medium (M) and large (L)—see Fig. 3. In the S setting, the kV imaging panel is lined up centrally with the tube and the FOV of 27.67 cm (at isocenter) is centered in the middle of the patient. For the M or L setting, the panel is moved up by 11.5 cm or 19 cm respectively, resulting in partial scans and allowing larger patient diameters to be scanned. The length of the field refers to the axial direction. The choices are 20 (nominal 26 cm), available for all three FOVs, and 10 (nominal 12.5 cm), for the M and L FOVs.

Several considerations contribute to the choice of length. Longer fields visualize more of the patient's anatomy for the alignment process, but they also deliver dose to a larger volume of the patient and may result in poorer image quality because of scatter from the larger beam. Interesting studies on the subject can be found in the literature.^(^ ^5^ ^,^ ^31^ ^)^

### B. Commissioning procedure

The commissioning of the Elekta Synergy kV imaging system comprised five categories of tests and establishment of a strategy for ongoing QA. The test categories, which are introduced in detail in the subsections that follow, were

1. system mechanical safety,
2. geometric accuracy (agreement of MV and kV beam isocenters),
3. image quality (resolution and low contrast visibility),
4. registration and correction accuracy, and
5. dose to patient.

Measures for ongoing QA are discussed following discussion of the individual tests.

#### *B.1 System mechanical safety*

The safety checks match closely with the procedures in the acceptance document provided by the manufacturer.

They consist of a check of all system interlocks (door interlock, kV source arm interlock, terminate key) and of all the system touch guards (accelerator head, kV imaging panel arm, MV imaging panel arm). To test the door interlock and the kV source arm interlock, an attempt is made to deliver X‐rays with either the door open or the kV source arm not fully extended. In addition the door interlock is tested by opening the door while X‐rays are being emitted. The terminate key is tested by pressing it while X‐rays are being emitted.

The touch guards at the various locations are tested by attempting to move the couch while triggering each guard separately.

#### *B.2 Geometric accuracy (agreement of MV and kV beam isocenters)*

Alignment of the isocenters of the kV imaging and the MV treatment system is crucial for accurate patient positioning, because the kV imaging system is used to position the patient with respect to the MV treatment system. The Elekta Synergy accelerator is based on a drum structure. The MV treatment (and imaging) system and the kV imaging system are mounted on arms of the drum structure. To account for the flexing that occurs in mechanical components of the imaging system arms and the accelerator beam arm at various gantry angles, the system uses a digital image correction with “flex maps.” The flex maps are lookup tables used to correct the images of the kV system for small deviations between the isocenters of the kV and MV systems, depending on gantry angle. These maps are initially created after installation of the system, and they are checked during preventive maintenance by a company service engineer. More details on how the geometric non‐idealities in the rotation of the gantry system are measured and corrected are provided in the literature.^(^ ^4^ ^,^ ^32^ ^)^

**Figure 4:**
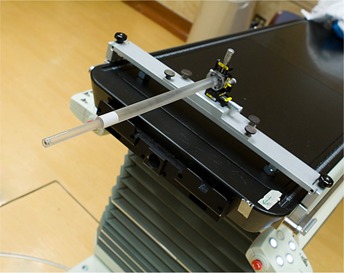
Ball bearing phantom. The phantom consists of a steel ball (diameter: 8 mm) located at the tip of a long plastic tube, which is connected to a base plate locked to the couch with a set of vernier adjustments that allow 0.01‐mm adjustments in the position of the steel ball.

The importance of the agreement of the isocenters warrants an additional check during commissioning. This check, which is also part of the manufacturer's acceptance testing, is performed using a ball‐bearing phantom supplied with the CBCT installation. The phantom consists of a steel ball (diameter: 8 mm) located at the tip of a long plastic tube, which is connected to a base plate locked to the couch with a set of vernier adjustments that allow the position of the steel ball to be adjusted in 0.01 mm increments (Fig. 4).

**Figure 5:**
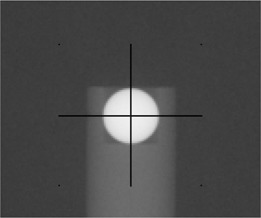
Sample kilovoltage image of the ball bearing phantom. The steel ball (diameter: 8 mm), which had previously been aligned with the center of megavoltage beam, is shown relative to the center of a kilovoltage image taken at one of the cardinal angles. Based on four such images (taken at all four cardinal angles), the maximum deviation of the kilovoltage isocenter from the megavoltage isocenter is determined.

The check comprises two steps:

- First, agreement between the location of the steel ball and the isocenter of the MV beam is established by image‐supported adjustment of the position of the ball. After initial setup using the in‐room laser system, eight MV images (port films) are taken at the four cardinal gantry angles and at collimator settings −90 degrees and 90 degrees. Based on analysis of the images by a system software routine, the deviation of the location of the ball from the location of the MV beam isocenter is calculated for x, y, and z. The position of the ball is adjusted, and the images are retaken. The process is repeated until the deviation in all three directions is below 0.25 mm, the threshold suggested by the manufacturer.
- In the second step, the deviation of the steel ball from the center of the kV imaging system is determined. Four images are taken with the kV system at the four cardinal gantry angles. On each image, pixel locations are used to determine the deviation between the center of the image (automatically marked with digital crosshairs by the software) and the center of the steel ball in the image (Fig. 5). In the highest magnification mode, with the digital crosshairs switched off, the cursor is positioned by the user at the observed center of the steel ball. The cursor location (horizontal and vertical) is displayed by the software once the corresponding tool is enabled. That location is then noted as the location of the steel ball for that image. Then, the crosshairs is switched on, and the same procedure is used to record its location. The horizontal and vertical differences between the observed center of the steel ball and the crosshairs are calculated for the image. These measurements and subtractions are performed for each of the four images. Based on other tolerances in the QA process of linear accelerators, the deviation limit for this test was set to 1.04 mm in each direction, which corresponds to 4 pixels. The kV image pixel size corresponds to 0.259 mm at the isocenter for this high‐resolution acquisition mode.

#### *B.3 Image quality*

The image quality achievable with the CBCT imaging system, consisting of X‐ray tube and flat panel, with the corresponding reconstruction algorithm, was tested for maximal achievable resolution and ability to display low‐contrast objects. Additional image quality measures, such as warping of the images, were assessed indirectly (see “Registration and correction accuracy” later in this paper) by their impact on the primary task of the system: patient alignment verification.

A Catphan phantom (The Phantom Laboratory, Salem, NY) was used for image quality measurements. The phantom consists of various cylindrical sections (modules), each of which is designed for a specific test. For the study, modules CTP528 (resolution measurements) and CTP404 (low‐contrast measurements) were used. The phantom containing these modules was scanned in the CBCT high‐resolution mode. The sections for spatial resolution (in the axial plane) and low‐contrast objects were evaluated in the XVI viewing software.

For spatial resolution, three observers determined the highest resolution line‐pair (lp) pattern for which the lines could be discriminated. The passing limit was set to the acceptance test specification of 7 lp/cm.

For the low‐contrast visibility test, the gray values of two cylindrical material samples in phantom section CTP404 were determined in the axial plane, where they are visible as circles. Using the image probe window, the mean and standard deviation of the gray values were recorded for polystyrene (PS, 1 o'clock position on the axial slice) and the low‐density polyethylene (LDPE, 3 o'clock position on axial slice). The window was set to a 40‐mm^2^ area, and 3‐slice averaging was selected. The measured numbers were combined to a percentage low contrast visibility by dividing the number 5.5 by the difference of the mean density values and then multiplying it by the arithmetic mean of their standard deviations. This procedure was adopted from the manufacturer's acceptance test, which calls for a resulting percentage less than 2%.

#### *B.4 Registration and correction accuracy*

The ability of the CBCT system to correctly register a localization geometry with a reference geometry was tested in two scenarios, using two different phantoms that went through the entire patient planning chain. Several methods of performing such tests have been presented in the literature.^(^ ^5^ ^,^ ^6^ ^,^ ^9^ ^,^ ^15^ ^)^ These methods were developed based on practical considerations such as availability of phantoms.

In the first test, the algorithm for repositioning the patient at the isocenter was tested to show that the system can precisely detect a preset deviation and determine the appropriate shift back to alignment. An anthropomorphic skull phantom (Model 603: CIRS, Norfolk, VA) was used for the test. With the CBCT system, alignment of the phantom on the treatment couch and its position relative to isocenter were first brought into agreement with a reference image, a previous CT scan. The phantom was then moved by specific distances in one or more directions relative to the reference position. Each movement of the phantom away from the initial start position was measured by the couch digital readout (1 mm resolution). The movements were each 10 mm in magnitude. The phantom was first moved “right,” then additionally “up,” and finally “in.” The ability of the XVI system to accurately describe these moves was analyzed for each step. At the end, the phantom was moved back to isocenter using the repositioning directions generated by the algorithm. Following the repositioning, another CBCT was taken and compared with the reference image.

In the second test, we investigated whether the algorithm could precisely describe shifts to a phantom for multiple locations throughout the phantom. This check can uncover potential distortions of the imaging and alignment process.

A Rando phantom (The Phantom Laboratory, Salem, NY) was loaded with plastic patient markers (Suremark, Simi Valley, CA) in four of its slices, which were separated from one another by one slice without markers. Three markers were embedded in each of the slices by removing the plugs and filling the hole with the marker and with bolus material (MedTec, Orange City, IA). Care was taken to eliminate air within the holes. The markers extended more than 15 cm along the superior–inferior axis and over more than a 9‐cm^2^ area within a slice. Using CBCT, the alignment of the Rando phantom on the treatment couch and its position relative to isocenter were first brought into agreement with a reference image, a previous CT scan. The phantom was then moved by 20 mm in each of the three dimensions (left, down, and out). These movements were considered to go well beyond expected patient misalignments and therefore to safely cover the range of patient adjustments likely to be encountered clinically.

The positions of all markers in the subsequent CBCT scan (localization image) were recorded with their x, y, and z coordinates. The positions of the markers in the CT scan (reference image) were also recorded. The marker positions were established as follows: with only one of the images visible at a time (reference or localization) for each marker the y coordinate (superior–inferior, see also Fig. 2) was determined by finding the most caudal and the most cranial slice (slice thickness: 1 mm) in which the marker was still visible and by calculating the mid‐position between them. At the closest axial slice to that position, the x and y coordinates were determined by visually analyzing the image of the marker and finding its center of gravity. Based on the thereby determined marker positions, deviations between the positions of the various markers in the CT set (reference image) and the CBCT set (localization image) were calculated and compared to the 20‐mm shifts that had been performed, as well as to deviations calculated by the system following the subsequent registration of the two images (automatic alignment algorithm for bone). The phantom was then moved according to the result of the registration, and another CBCT was performed. The locations of the markers in the now aligned localization image were established and compared to those in the reference image.

#### *B.5 Dose to patient and dosimetric stability*

The dose to the patient from a CBCT depends significantly on the scanning parameters used. Additionally, the dose across the patient may vary, depending on scanning technique. For instance, in the M and L settings, some areas of the patient will be irradiated during only parts of a 360‐degree scan, but the core of the scan will be irradiated at all times.

Patterns of dose distribution, and the impacts of the various factors on the dose, present opportunities for exciting research, some of which has already been reported by others.^(^ ^5^ ^,^ ^13^ ^,^ ^33^ ^)^

With respect to patient dose, the objective of QA is to establish a baseline measure of patient dose and to monitor that measure over time to ensure that patient dose does not increase. The measure needs to be reproducible and closely related to dose. The combination of an air kerma measurement for a 360‐degree CBCT and measurement of the half‐value layer of the kV beam as a description of beam quality fulfills this requirement.

Both measurements were performed for a commonly used beam energy (120 kV). The air kerma was measured using a Farmer chamber that had been calibrated in the kV energy range by an accredited laboratory. The chamber was held off the end of the treatment couch at isocenter. Repeated measurements were performed using the S20 insert and 120 kV, 40 mA, and 40 ms per frame for a 360‐degree scan (~620 frames). These numbers were based on a parameter preset by the manufacturer, and they represent the highest load used in the initial CBCT imaging at our department.

The half‐value layer of the kV beam was determined using a stationary beam with aluminum plates and standard procedures.^(34)^

## III. RESULTS

### A. Commissioning procedure

#### *A.1 System mechanical safety*

The beam termination at the control console and the door interlock worked correctly. A not‐fully‐extended kV source arm and an open door each inhibited engagement of the beam. All touch guards were found to work correctly, in that they inhibited motion when activated.

#### *A.2 Geometric accuracy (agreement of MV and kV beam isocenters)*

After three iterations, the maximal deviation of the steel ball location from the MV isocenter location was below the threshold of 0.25 mm.

Analysis of the subsequent kV images determined that the maximal deviation between the kV isocenter and the steel ball was 0.5 mm or less in each orthogonal direction. Fig. 5 shows an example of the steel ball aligned with the center of one of the kV images.

#### *A.3 Image quality*

The system was found to be able to resolve 9 lp/cm. Low‐contrast visibility of 1.82% was measured.

#### *A.4 Registration and correction accuracy*

The results of this test, summarized in Table 1, show the initial positional deviations of the anthropomorphic skull phantom about the linear accelerator isocenter after alignment to the previous CT scan as “Step 1.” Steps 2 – 4 are the cumulative shifts of 10 mm each in the x direction (Step 2, patient's right), the z direction (Step 3, couch up), and the y direction (Step 5, couch in).

The data in the corresponding columns on the right show that the algorithm detected the translational shift for every move with an accuracy of better than 1 mm, which is the equivalent of the tolerance of the couch. The rotational error (which should be zero, because no rotations were introduced) stayed within 0.6 degree for this part of the test.

After Step 4, two more scans and image alignments were performed without moving the phantom to verify the reproducibility of the system. They are noted as Steps 5 and 6 in Table 1. The fluctuation found in this test was within 0.5 mm and 0.5 degree for translation and rotation respectively.

**Table 1:** Position errors reported by the registration algorithm (“automatic bone” mode) following defined moves of the phantom

Step	Defined move of phantom		Position error after “automatic bone” alignment	
			Translation (mm)	Rotation (degrees)
1	Starting point	X	0.5	360.0
		Y	0.2	360.0
		Z	0.3	0.0
2	X: −10 mm	X	−9.2	0.0
	(1 cm patient's right)	Y	−0.1	0.2
		Z	0.4	0.0
3	Z: 10 mm	X	−9.1	0.1
	(additional 1 cm up)	Y	0.0	0.1
		Z	10.3	0.6
4	Y: 10 mm	X	−9.2	360.0
	(additional 1 cm couch in)	Y	9.5	0.1
		Z	10.2	360.0
5	None	X	−9.2	360.0
	(reproducibility check)	Y	9.8	0.2
		Z	10.7	0.5
6	None	X	−9.1	360.0
	(reproducibility check)	Y	9.4	360.0
		Z	10.3	360.0
7	x: 9.1 mm; y: −9.4 mm; z: −10.3 mm	X	0.0	360.0
	(corrections suggested by system to return to agreement)	Y	0.7	359.0
		Z	0.8	0.5

**Figure 6:**
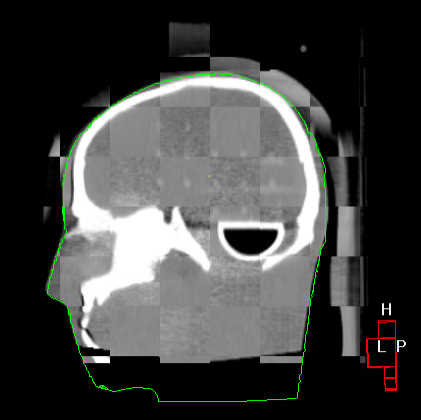
Sagittal view of the head phantom after registration. The selected display type is “cut,” showing alternating panels of reconstructed and reference image.

In the final step, Step 7, the phantom was moved back to isocenter using the repositioning directions generated by the algorithm. The data show that the phantom was brought within 0.5 mm and 0.5 degree of the starting point (Step 1). Fig. 6 shows a sagittal view of the phantom after registration. The selected display type is “cut,” showing alternating panels of reconstructed and reference image.

Based on the foregoing tests, we concluded that the accuracy of the system for repositioning deviations is equal to or better than 1 mm and 1 degree in each dimension.

The analysis of the x, y, and z coordinates (Fig. 2) for 12 markers within the Rando phantom on the CBCT localizing scan, taken after the phantom had been shifted 20 mm out, 20 mm left, and 20 mm down from an aligned position, demonstrated the capability of the system to identify those shifts. The average detected shifts in the x, y, and z direction were −20.35 mm (SD: 0.73 mm), 20.33 mm (SD: 0.72 mm), and 19.30 mm (SD: 0.58 mm) respectively. Using the bone‐based automatic alignment tool, the system suggested corrective shifts of 20.8 mm (x), −20.5 mm (y), and −19.7 mm (z). The system also suggested rotations of 0.0, 0.6, and −1.0 degree around the x, y, and z axes respectively.

After the suggested shifts had been performed, the average agreement for the 12 markers in the x, y, and z coordinates was 0.1 mm (SD: 0.21 mm), −0.12 mm (SD: 0.55 mm), and 0.22 mm (SD: 0.21 mm) respectively. The largest deviations found among these points were 0.6 mm, 1.0 mm, and 0.5 mm for the x, y, and z coordinates respectively.

#### *A.5 Dose to patient and dosimetric stability*

The air kerma was measured as 6.28 cGy (SD: 0.034 cGy) with S20 FOV, 120 kV, 40 mA, 40 ms per frame, for a 360‐degree scan (~620 frames).

Additional measurements also showed that the kerma scales linearly with milliamperes. The half‐value layer of the beam was found to be 7.1 mm Al.

### B. Implementation of ongoing QA

The first three categories of tests—system mechanical safety, geometric accuracy (agreement of MV and kV beam isocenters), and image quality (resolution and low‐contrast visibility)—are performed on a monthly basis as part of the machine QA procedure. The pass criterion for the isocenter alignment is 1 mm, as described in “Methods,” above. For the monthly image quality checks, the image quality found during this commissioning procedure are used as a baseline. The pass criteria for spatial resolution and low‐contrast visibility are ≥7 lp/cm and ≤2% respectively.

The monthly QA performed over 8 months shows that the system always passed all three tests. The average deviation in agreement between the MV and kV beam isocenters in all directions was less than 0.42 pixel (~0.11 mm), with a maximum of 3 pixels (~0.78 mm). In the image quality tests, the detected spatial resolution ranged between 8 lp/cm and 9 lp/cm, and the low‐contrast visibility was found well below the threshold of 2% at 1.1% (average).

The other two tests of the acceptance procedure—registration and correction accuracy, and dose to patient and dosimetric stability—will be repeated annually, because the possibility of change in that part of the system is smaller. Pass criteria for the registration and correction accuracy is 1 mm or 1 degree in each dimension. The patient dose must not increase by more than 5%, a reasonable estimation of the uncertainty of the initial measurement.

All tests will also be performed in cases of system changes, such as replacement of major parts, including the X‐ray tube or the imaging panel.

**Figure 7:**
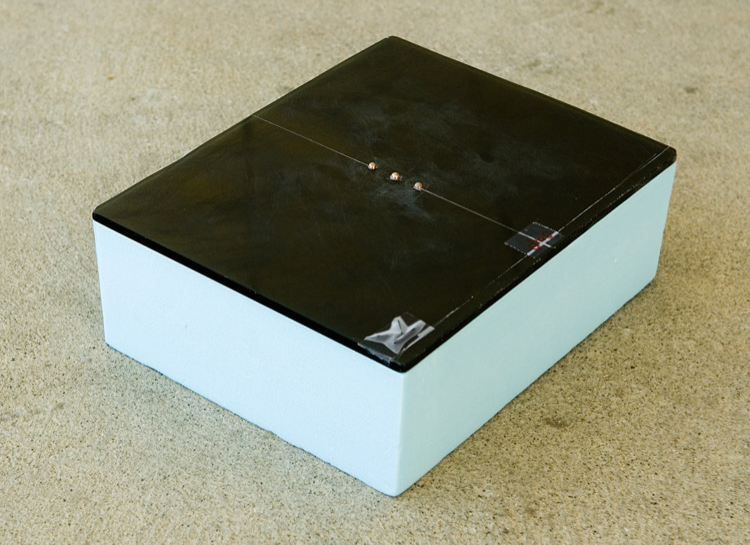
Morning quality assurance phantom. Consisting of three small steel balls (BBs) mounted half‐sunken in a Lucite plate on a Styrofoam base, this phantom is used verify agreement of the isocenters of the kilovoltage and megavoltage beams.

In addition, a daily QA procedure has been implemented to verify agreement of the isocenters of the kV and MV beams. A simple phantom consisting of three small steel balls (BBs) mounted half‐sunken in a Lucite plate on a Styrofoam base (Fig. 7) is used. As part of the morning QA, the phantom is aligned to the crosshairs and imaged at two angles (anterior–posterior and lateral) with both the kV and the MV imaging system. Agreement between the positions of the BBs in the kV and MV images is checked using the graticule placed in the MV beam line and the electronic graticule for the kV imaging line. Emphasis is placed on agreement of the locations in both image sets rather than on the absolute position of the balls in either image. A deviation of 2 mm in each direction is allowed. The daily QA procedure is also performed during monthly QA to tie it to the more precise isocenter agreement verification performed at that time. The frequency of the daily QA procedure check will be reevaluated based on the data collected during approximately 1 year.

## IV. DISCUSSION

With respect to QA, CBCT presents a new challenge to the clinical medical physicist. Because positioning of the patient is based directly on data from the CBCT systems, their performance has a major effect on the outcome of treatment. The accuracy and reliability of CBCT systems therefore need to be tested and related to familiar tolerances of the treatment process.

The set of tests presented here was performed on one of the first commercially available kV imaging–based CBCT systems in clinical use. The set is comprehensive and at the same time mindful of the time pressures on the physicist. More extensive testing of the CBCT system may be justified with continued clinical use, but the described tests represent clinical reality and ensure that the performance of the system is maintained at acceptable levels.

After basic system safety, a significant point of concern is the agreement of the kV imaging line isocenter and the MV treatment line isocenter. In the commissioning, this agreement was found to be within 0.75 mm. Because this parameter is potentially as important as machine output, a daily QA procedure was established to monitor agreement of the isocenters. The importance of this agreement has already been reported by others,^(15)^ and recently a commercial phantom became available (Penta Guide: Modus Medical Devices, London, ON, Canada).

Two components that are likely to suffer at least some degree of degradation in ongoing use are the X‐ray tube and the corresponding imaging panel. Over time, depositions inside the vacuum tube will change the beam quality of the kV beam. The imaging panel will be exposed to direct radiation during kV imaging and to scatter radiation from the MV or electron beam during treatment. Changes in its performance over time are conceivable, and therefore monitoring of image quality is needed. The suggested monthly measurement of resolution of line pairs per centimeter, together with the described low‐contrast visibility test, is one approach for such monitoring. With the expanding role of imaging not only in treatment planning but also in treatment delivery, the need for the radiotherapy medical physicist to understand imaging quality parameters increases as does the need for high‐quality imaging phantoms in radiotherapy departments.

Image registration to evaluate the patient's position relative to the planning position is a new component of the treatment workflow in radiation therapy. The algorithms involved therefore deserve special attention, and QA is likely to change over time as experience is gathered. As noted earlier, several QA approaches and methods have been reported in the literature.^(^ ^5^ ^,^ ^6^ ^,^ ^9^ ^,^ ^15^ ^)^ The two tests performed in our commissioning procedure convinced us of the current fitness of the machine. In the first test, using a skull phantom, the image analysis and position correction algorithm was found to be within the resolution of the patient couch motion (1 mm and 1 degree). In the more comprehensive second test, comparing coordinates of 12 markers in a Rando phantom that had been moved 20 mm in each direction in a registered CBCT scan to coordinates in the reference CT, the average deviation in the x, y, and z coordinates was well below 1 mm. The largest deviations found among these points were 0.6 mm, 1.0 mm, and 0.5 mm respectively. The larger deviation for the y component can be partly attributed to the slice thickness of 1 mm, which is along the y direction in the system's coordinate system. Although this excellent agreement was obtained with a rigid phantom and will likely not directly translate to a patient, it nevertheless shows the accuracy of the algorithm throughout a large volume.

The accuracy results obtained agree with the literature. One group found in their phantom studies that “sub–pixel size set‐up errors (down to 0.5 mm) can be correctly determined.”^(6)^ Another very detailed study demonstrated that the XVI system was capable of positioning “an unambiguous object to within less than 1 mm of the prescribed location.”^(15)^

The dose to the patient from a CBCT scan is an interesting field of research—in particular its relation to image quality. If the primary use for the images is to enable reliable patient alignment before treatment, image quality need be sufficient only for that task. Our initial experience shows that fractions of the dose (starting at the manufacturer's provided default values and measured as reduced milliamperes)^(35)^ are often sufficient. A systematic study using the XVI scans and another registration algorithm found that a scan with “ultra low dose” led to the same alignment (within 0.1 mm and 0.1 degrees) despite reduced subjective image quality than a more “regular” scan involving higher patient dose.^(9)^ Optimal settings of milliampere field size and other parameters specific to each anatomic area will be found through clinical use and protocol development.

The use of CBCT scans for dose recalculation based on the patient's anatomy on the day of treatment is an appealing application. The correct and patient‐size independent representation of Hounsfield units remains a general problem at this point.^(36)^ Also, checks beyond those presented here will need to be performed to ensure the fitness of a particular CBCT system for that task.

Ongoing QA plays an important role in implementation of the new IGRT technologies, because some of the tools are first‐generation devices that require special attention. The proposed continuing QA methods are believed to be adequate to ensure safe operation of CBCT. With more experience, some tests may be able to be simplified or to have their frequency reduced. However, QA of this new capability will always be needed, because it involves technical components that can potentially malfunction. New tools will likely help to make the QA process more efficient. Unlike the situation with intensity‐modulated radiotherapy (IMRT), the last major development in the field, no obvious need for patient‐specific QA seems to arise with IGRT (not to be confused with patient‐specific use of the tools of IGRT—that is, adjustment of imaging parameters and alignment methods, which is called for from the beginning).

## V. CONCLUSIONS

Image‐guided radiotherapy is an exciting new chapter for radiation oncology. With CBCT, patients can be objectively and precisely positioned for treatment, matching the images used for treatment planning with those taken just before the treatment session. At the same time, IGRT systems open the door to new areas of research, with the goal of optimizing patient care and achieving cure. The described procedures allow for safe clinical implementation of CBCT systems in a modern radiotherapy department.

**Table 2:** Attachment 1: Monthly quality assurance worksheet

Cone‐beam CT QA for the month of:__________________		
month / year		
Performed:	Date:	Initials:
Approved:	Date:	Initials:

### *1. Physical operation and safety*

**Table:** 

System interlocks	Interlock	Check result
To test the door interlock and the kV source arm interlock, an attempt is made to deliver X‐rays with either the door open or the kV source arm not fully extended. In addition the door interlock is tested by opening the door while X‐rays are emitted. The latter is also performed for the Terminate key.	Door	□ Prevent □ Stop
	kV source arm	□ Prevent
	Terminate key	□ Stop

Touch guards	Equipment	Touch guards check results
The touch guards at the different locations are tested by attempting to move the couch while triggering each of them separately.	Digital accelerator	□ Head
	kV imaging arm	□ Panel □ Middle arm
	iViewGT imaging arm	□ Panel □ Middle arm

## *2. kV–MV isocenter alignment verification*

The check is performed with the ball bearing phantom. **Step 1:** Agreement between the location of the steel ball and the isocenter of the MV beam is established by image‐supported adjustment of the position of the ball. After initial setup using the inroom laser, eight MV images (port films) are taken at the four cardinal gantry angles at collimator settings ‐90 and 90 degree. Based on the analysis of the images using a routine in the XVI system software, the deviation of the location of the ball from the location of the MV beam isocenter is calculated for x, y and z. If the deviation in one of the three directions is larger than 0.25 mm, the position of the ball is adjusted and the images are retaken. This process is repeated until the deviation in all three directions is below 0.25 mm. **Step 2:** The deviation of the steel ball, which now represents the isocenter of the MV beam, and the kV imaging system is determined. Four images of the four cardinal gantry angles are taken with the kV system. The deviation between the center of the image and the center of the steel ball in the image is determined and reported for each of the images.

**Table:** 

		Center of ball bearing		Image center		Difference	
Image number	Gantry angle	Horizontal	Vertical	Horizontal	Vertical	Horizontal	Vertical
1	0°	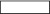	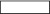	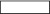	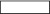	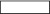	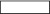
2	90°	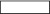	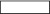	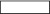	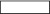	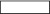	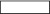
3	$-90^{{}^{\circ}}$	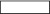	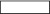	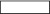	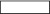	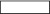	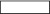
4	180°	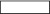	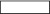	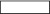	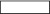	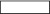	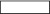

The maximal deviation should be 4 pixels (1.04 mm) or less in each orthogonal direction.

## *3. Image quality for the kV system*

The CATPHAN phantom is used for these measurements. The phantom is scanned in the high resolution mode in the service section of the XVI software. The sections for spatial resolution (in the axial plane) and low contrast objects are evaluated in the XVI viewing software. For the spatial resolution the observer evaluates which is the highest resolution line pair pattern for which the lines can be discriminated from each other.

Resolution test: 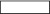 lp/cm *The resolution should be at least 7 lp/cm*.

For the low contrast visibility test the gray value of the Polystyrene sample (PS – 1 o'clock position on axial slice) and the Low Density Polyethylene sampe (LDPE – 3 o'clock position on axial slice) are measured in the CTP 404 module of CATPHAN. The window is set to a 40 mm^2^ area and 3 slice averaging is selected. Record the mean and standard deviation of the gray value in the following table and calculate percentage low contrast visibility (LCV):

$$LCV=\frac{2.75\cdot (SD_{PS}+SD_{LDPE})}{MEAN_{PS}-MEAN_{LDPE}}\;\;\;\%$$

The LCV needs to be smaller than 2%.

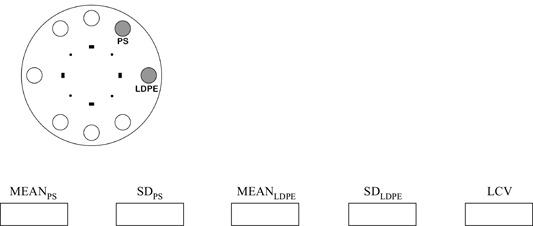

## Supporting information

Supplementary Material
