# Supplementary figures and images for: Commissioning experience with cone‐beam computed tomography for image‐guided radiation therapy
Source: J Appl Clin Med Phys. 2007 Jul 17;8(3):21–36. doi: 10.1120/jacmp.v8i3.2354 (PMC5722599; doi:10.1120/jacmp.v8i3.2354)

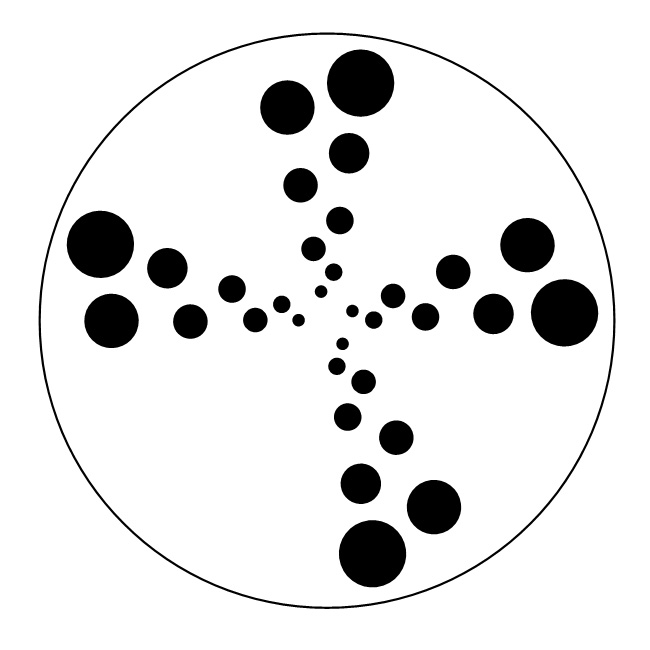

Supplement: Supplementary file 1 — Supplementary Material [file ACM2-8-021-s001.jpg]
